# Analysis of Cortico-Muscular Coupling and Functional Brain Network under Different Standing Balance Paradigms

**DOI:** 10.3390/brainsci14010081

**Published:** 2024-01-13

**Authors:** Weijie Ke, Zhizeng Luo

**Affiliations:** Institute of Intelligent Control and Robotics, Hangzhou Dianzi University, Hangzhou 310018, China; ksg233@163.com

**Keywords:** standing balance, EEG/EMG, cortico-muscular coupling, functional brain network, neuromuscular control

## Abstract

Maintaining standing balance is essential for people to engage in productive activities in daily life. However, the process of interaction between the cortex and the muscles during balance regulation is understudied. Four balance paradigms of different difficulty were designed by closing eyes and laying sponge pad under feet. Ten healthy subjects were recruited to stand for ten 15 s trials in each paradigm. This study used simultaneously acquired electroencephalography (EEG) and electromyography (EMG) to investigate changes in the human cortico-muscular coupling relationship and functional brain network characteristics during balance control. The coherence and causality of EEG and EMG signals were calculated by magnitude-squared coherence (MSC) and transfer entropy (TE). It was found that changes in balance strategies may lead to a shift in cortico-muscular coherence (CMC) from the beta band to the gamma band when the difficulty of balance increased. As subjects performed the four standing balance paradigms, the causality of the beta band and the gamma band was stronger in the descending neural pathway than that in the ascending neural pathway. A multi-rhythmic functional brain network with 19 EEG channels was constructed and analyzed based on graph theory, showing that its topology also changed with changes in balance difficulty. These results show an active adjustment of the sensorimotor system under different balance paradigms and provide new insights into the endogenous physiological mechanisms underlying the control of standing balance.

## 1. Introduction

Standing balance refers to the ability of the human body to automatically adjust and maintain a stable posture in a resting state. A degeneration of the musculoskeletal system, as well as neurological damage from stroke, can affect an individual’s ability to balance [1,2]. Maintaining standing balance is a complex physiological process that involves the coordinated work of the nervous system, musculoskeletal system, and sensory system. The nervous system integrates vestibular, proprioceptive, and visual information received by the sensory system and sends instructions to the muscular system in the form of bioelectrical signals to achieve control over the balance of the body [3]. At present, there are various evaluation methods for standing balance [4,5], which provide some objective indicators, but there is relatively little research on the internal physiological mechanism of standing balance regulation.

When a person is in active motion, the interaction between the electroencephalography (EEG) signals generated by the cerebral cortex and the electromyography (EMG) signals generated by the limb muscles in different rhythms is called the cortico-muscular coupling. Neurotransmission and proprioceptive feedback in the process of motor control are organically integrated. Researchers usually use cortico-muscular coherence (CMC) to analyze synchronicity between the cerebral cortex and the muscles [6,7]. Alkaff et al. [8] found more significant CMC in a one-legged stance task than in a bipedal stance task, suggesting that the interaction between the cerebral cortex and the muscles of the lower limbs was strengthened to compensate for postural instability during the one-legged stance task. Spedden et al. [9] found that compared with young people, older people had lower cortico-, intra-, and intermuscular coherence in the beta band when performing a task of static contraction of the lower limbs. Bao et al. [10] recruited 10 stroke patients for neuromuscular electrical stimulation pedaling interventions and found that rehabilitation training promoted the interaction between the ipsilateral hemisphere and the lower limb during isometric contraction, indicating that CMC has potential utility in understanding neuromuscular changes. Quantitative analysis of CMC under different balance paradigms helps us to gain insight into the physiological correlation of the neuromuscular system. Similarly, this correlation is also reflected in the flow of information (i.e., causality). There exists a bidirectional functional connectivity between the cortex and muscles [11], namely descending (from cortex to muscle) and ascending (from muscle to cortex) neural pathways. Although CMC is of obvious value in cortico-muscular synergy analysis, it cannot be used to describe and infer the direction of information flow. Transfer entropy (TE) is an information-theoretic measure used to measure causality. Because it does not depend on its own system history, it is often used in the analysis of nonlinear system models [12]. TE has unique advantages in the analysis of bioelectrical signals. It can be used not only to untangle complex relationships between signals, but also to analyze the functional connection and information transmission of biological systems [13,14].

As the most complex organ in the human body, the brain is composed of tens of billions of neurons. These neurons communicate dynamically, forming an intricate network structure [15]. Brain waves are generated by neuronal activity in the brain and are typically categorized into five types based on their frequencies: delta waves typically occur during deep sleep; theta waves mainly appear when a person is tired or drowsy; alpha waves decrease with increased eye activity; beta waves are abundant during focused attention and active thinking; gamma waves are generally considered to be associated with higher cognitive processes [16]. The completion of various movements requires the cooperation of multiple brain regions. Therefore, modeling the cerebral cortex using complex network theory is beneficial for dissecting the behavior and mechanism of the cerebral cortex. Network characteristics such as the average clustering coefficient and average characteristic path length can better reflect the closeness of coordination between brain regions [17,18]. However, the impact of different balance paradigms on brain network connectivity also needs to be further studied.

In this paper, four balance paradigms of different difficulty were designed by closing eyes and laying sponge pad under feet to explore the endogenous physiological mechanisms underlying the control of standing balance. We analyzed the balance control strategies of the neuromuscular system using CMC and TE. In addition, a multi-rhythmic functional brain network was constructed to dig deeper into the neural mechanism of balance regulation. This work provides a new perspective for exploring the balance control mechanism of the central nervous system and evaluating the motor rehabilitation of patients with impaired standing function.

## 2. Materials and Methods

### 2.1. Framework

**Figure 1:**
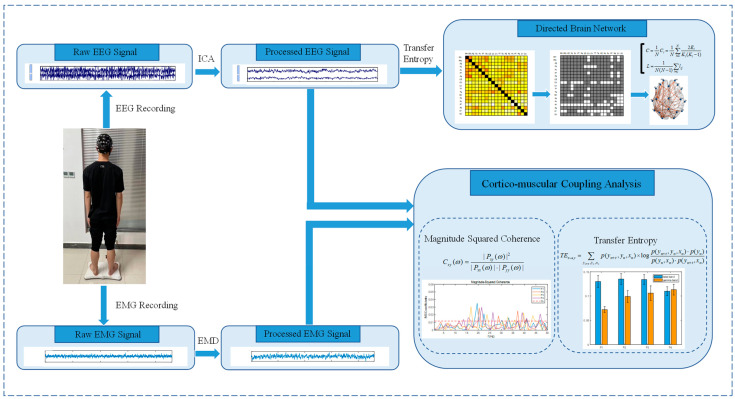
Experimental framework.

This section briefly introduces the general idea of this study. First, EEG and EMG signals were acquired synchronously. Second, EEG and EMG data were preprocessed to remove noise. Then, CMC and TE were used to analyze functional cortico-muscular coupling and transmission. Studies have shown that there is coherence between the EEG channel CZ and the EMG of distal muscles of the lower limbs [19], so we chose the EEG channel CZ to calculate the strength of the cortico-muscular coupling with lower limb muscles. Finally, a functional brain network was constructed to analyze the network characteristics of standing balance regulation. The general framework of the experiment is shown in Figure 1.

### 2.2. Experimental Subjects and Experimental Paradigms

**Figure 2:**
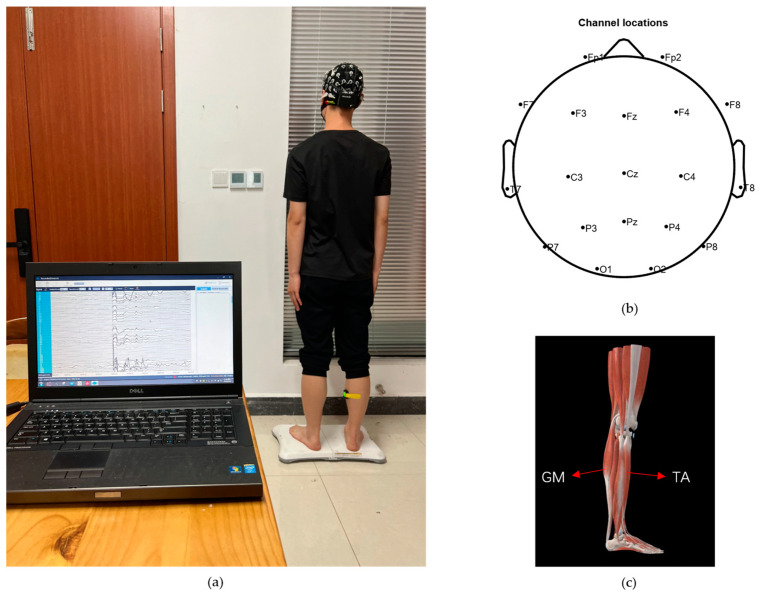
Experimental process. (**a**) Experimental environment, (**b**) EEG electrode arrangement, and (**c**) muscle distribution.

Ten healthy nonathlete subjects (7 males and 3 females; mean ± standard deviation, age = 23.0 ± 0.6 year; height = 172 ± 7.3 cm; weight = 66.1 ± 10.8 kg) were recruited. They understood and signed an informed consent form in accordance with the Declaration of Helsinki before the experiment and complied with the following requirements within three days before the experiments: (1) no strenuous exercise, (2) clean scalp and hair, (3) adequate sleep, (4) no consumption of stimulating drinks. The experimental environment is shown in Figure 2a.

Maintaining standing balance is a closed-loop control process that requires the central nervous system to receive vestibular, proprioceptive, and visual information to control muscle contraction to overcome disturbances in the external environment and ultimately stabilize the body. When peripheral sensory feedback decreases, it becomes more difficult for the human body to maintain postural stability. Due to the lack of an experimental paradigm that can block the vestibular sense in humans, we merely blocked visual input and proprioceptive input from the soles of the feet by having the participants close their eyes and laying sponge pads under their feet, respectively. The four balance paradigms are shown in Table 1. A 64-lead EEG acquisition instrument (Neusen. W64, Neuracle, Changzhou, China) was used to obtain EEG signals, and the sampling frequency was set to 1000 Hz. Before data acquisition, the detection electrodes needed to be prepared with conductive paste to make the impedance lower than 5 kΩ. The electrodes were distributed according to the 10–20 international standard. Data from 19 channels (FP1, FP2, FZ, F3, F4, F7, F8, CZ, C3, C4, T7, T8, PZ, P3, P4, P7, P8, O1, and O2) were selected for analysis. These selected electrodes are representative of the somatosensory and motor areas [20]. Channel distribution is shown in Figure 2b. The EMG acquisition device (Delsys Inc., Natick, MA, USA) is a wireless surface EMG sampling system, with a sampling frequency of 2148 Hz., The epidermis over each muscle of interest was wiped with alcohol before data collection. EMG signals were acquired from the gastrocnemius (GM) and tibialis anterior (TA). These muscles exhibit high levels of activity during the process of posture adjustment [21]. The location of the muscles is shown in Figure 2c.

Hytönen et al. [22] recruited 212 healthy volunteers of different ages, quantified the impact of vision and proprioception on their postural stability, and found that proprioception is more critical than vision for balance control in young people. Maintaining standing balance is most difficult when both vision and proprioception are blocked. Therefore, this paper identifies the balance difficulty of the four paradigms as P4 > P3 > P2 > P1.

**Figure 3:**
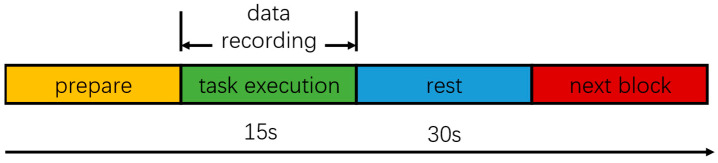
Experimental design.

Each subject stood on the balance board with their legs shoulder-width apart and parallel, with both hands hanging down naturally beside their thighs. The synchronous collection of experimental data began when the subject was able to stand stably. Each subject completed the tasks of the P1–P4 in order. For each paradigm, the experiment required the synchronous collection of EEG and EMG for 15 s. Each paradigm required the subject to perform 10 repetitions, and after each period of data collection, the subject took a 30 s rest period to avoid muscle fatigue. In the P1 and P3 paradigms, the subject was instructed to look straight ahead at a reference object with both eyes. If any event, such as a fall or a cough, interrupted data collection during the experiments, the experimental data were invalidated, and the test was repeated after a period of rest. The experimental process is shown in Figure 3. In addition, the height of the sponge pad was 10 cm and its density was 30 ${\mathrm{kg}/m}^{3}$.

### 2.3. EEG/EMG Signal Preprocessing

EEG data are typically divided into 5 functional frequency bands: delta (1–4 Hz), theta (4–8 Hz), alpha (8–13 Hz), beta (13–30 Hz), and gamma (30–50 Hz) [23]. The rhythmic activity of each frequency band corresponds to a different cortical neural activity. Since EEG and EMG are noninvasive data acquisition techniques that contain considerable noise, data preprocessing is needed. The toolbox EEGLAB version 2019 was chosen to preprocess the raw EEG data. EEG processing included a 1~50 Hz bandpass filter with a 50 Hz power frequency notch as well as independent component analysis to remove artifacts of eye movement or muscle movement [24]. For EMG signals, we first applied a 50 Hz power frequency notch, then used empirical mode decomposition to denoise the data; finally, we downsampled the signals to 1000 Hz.

### 2.4. Magnitude-Squared Coherence

MSC is a method used to measure the degree of linear relationship between two signals at a specific frequency. It is widely used in the analysis of biological signals [23,25]. Its mathematical expression is as follows:

(1) $$C_{xy}(\omega )=\frac{|P_{xy}(\omega ){|}^{2}}{\left|P_{xx}(\omega )|\cdot |P_{yy}(\omega )\right|}$$

where *x* and *y* are the EEG signal and EMG signal to be analyzed, respectively. Equation (4) represents the coherence coefficient of signal *x* and signal *y* at frequency ω. $P_{xy}(\omega )$ represents the cross-power spectral density of signal *x* and signal *y* at frequency ω. $P_{xx}(\omega )$ and $P_{yy}(\omega )$ represent the self-power spectral density of signal *x* and signal *y* at frequency ω.

### 2.5. Coherence Threshold Evaluation

To calculate the significant coherence area, which measures the degree of coupling of EEG and EMG signals, it is necessary to judge whether the MSC results are statistically significant [26]. The coherence coefficient threshold *CL* is introduced as follows:

(2) $$CL=1-{\left(1-\alpha \right)}^{\frac{1}{T-1}}$$

where α represents the confidence level, which is usually set to 0.95, and *T* represents the number of data points participating in the Fourier transform used in the coherence calculation process. *T* was set to 256 in this study. If the value of the coherence coefficient is greater than *CL*, it means that the coherence between the EEG and EMG signals is significant at frequency ω; otherwise, it means that the coherence between the EEG and EMG signals is not statistically significant at frequency ω.

The significant coherence area is defined as the area between the coherence threshold *CL* and the coherence curve, which is calculated as follows:

(3) $$A_{coh}=\sum_{\omega }\Delta \omega (S_{co}(\omega )-CL)$$

where Δω represents the frequency resolution and $S_{co}(\omega )$ represents the value of the coherence coefficient greater than the coherence threshold *CL* at frequency ω. The coherence of EEG and EMG signals in a certain frequency band is positively correlated with the significant coherence area.

### 2.6. Transfer Entropy

In 2000, Schreiber [27] proposed transfer entropy (TE) to quantify the information flow between stochastic systems. Given two signals *x* and *y,* representing EEG and EMG, respectively, TE from *x* to *y* is calculated as follows:

(4) $$\begin{array}{r} TE_{x\to y}=\sum_{y_{n+\tau },y_{n},x_{n}}p(y_{n+\tau },y_{n},x_{n})\times \log\\ \frac{p(y_{n+\tau },y_{n},x_{n})\cdot p(y_{n})}{p(y_{n},x_{n})\cdot p(y_{n+\tau },x_{n})} \end{array}$$

where $p(y_{n+\tau },y_{n},x_{n})$ represents the joint probability density between variables and τ denotes the predicted delay time of the two signals. In this study, τ was set to 26 and 29 for the descending and ascending neural pathways, respectively [11]. 

### 2.7. Graph Theory

By calculating the value of TE between 19 EEG channels, a 19 × 19 weighted directed matrix *m* can be obtained. The weighted directed matrix is binarized using the cost efficiency threshold, which removes the influence of weakly weighted edges. The cost efficiency threshold δ is calculated as follows:

(5) δ=max{Ce}=max{G−D}

where *D* represents network density, which is defined as the ratio of the actual number of edges in the network to the maximum possible number of edges; *G* is the global efficiency of the network, and its expression is as follows:

(6) $$G=\frac{1}{N(N-1)}\sum_{i\neq j}^{N}\frac{1}{l_{i,j}}$$

where *N* represents the number of nodes in the network, while $l_{i,j}$ represents the shortest path from node *i* to node *j*.

If TE from node *i* to node *j* is less than δ, then $m_{ij}$ is equal to 0; otherwise, $m_{ij}$ is equal to 1.

(7) $$m_{ij}=\left\{\begin{array}{l} 0,{\;\mathrm{TE}}_{i\to j}<\delta \\ 1,{\;\mathrm{TE}}_{i\to j}\geq \delta \end{array}\right.$$

The clustering coefficient ($C_{i}$) of node *i* describes the probability that any pair of nodes in the neighbor nodes of node *i* has an edge connection. The average clustering coefficient (*C*) of the network is the mean of the sum of the clustering coefficients of all nodes, and the average characteristic path length (*L*) is the average of the shortest paths between all node pairs. Both *C* and *L* are indicators to measure the connection relationship and closeness between network nodes. *C* is positively correlated with the connectivity of the network topology, while *L* is negatively correlated with the connectivity of the network topology.

(8) $$C=\frac{1}{N}C_{i}=\frac{1}{N}\sum_{i=1}^{N}\frac{2E_{i}}{K_{i}(K_{i}-1)}$$

(9) $$L=\frac{1}{N(N-1)}\sum_{i\neq j}l_{ij}$$

where $E_{i}$ is the actual number of edges between the neighbor nodes of node *i*, and $K_{i}(K_{i}-1)/2$ is the maximum number of edges that may exist among these neighbor nodes.

### 2.8. Statistical Analysis

The paired sample t-test was used to evaluate the differences in *C* and *L* between P1 and P2, P1 and P3, P2 and P4, and P3 and P4. In this paper, the confidence level was set to 0.05 to judge whether there is a significant difference between the data.

## 3. Results

### 3.1. CMC Analysis

To study the regulatory information exchanged between the cerebral cortex and lower limb muscles in the process of balance control, the EEG signal of the CZ channel, which is more related to the control of lower limb muscles, was selected to calculate the coherence coefficient with the EMG signals of GM and TA and analyze the coherence changes of EEG and EMG in different frequency bands as the human body maintains balance in a standing position.

**Figure 4:**
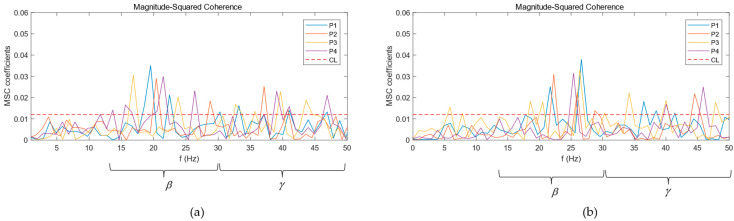
MSC curves between EEG and EMG: (**a**) MSC curves of TA; (**b**) MSC curves of GM.

Figure 4 show the coherence curves of EEG and EMG of Subject S1 under the four balance paradigms. The horizontal dotted line is the coherence threshold *CL*. If the coherence coefficient curve is above the dotted line, it means that EEG and EMG are significantly coherent in this frequency range. It can be seen that the significant coherence area was mainly concentrated in the beta band and gamma band; accordingly, the mean of the significant coherence area of the EEG and EMG of ten subjects in the beta band and gamma band are calculated according to Formula (3) to quantitatively analyze the coherence changes of EEG and EMG under different balance paradigms. The statistics of the means of the significant coherence areas are shown in Table 2. By comparing the significant coherence area, it was found that the coherence of the beta band under the four balance paradigms was more significant than that of the gamma band. In the gamma band, under the two balance paradigms with single sensory input blockade, P2 and P3, the significant coherence area increased significantly compared with the P1 paradigm, while under the double sensory input blockade, P4, the significant coherence area increased the most compared with P1. However, the significant coherence area of the beta band under P2, P3, and P4 reduced compared with P1.

In addition, the significant coherence frequency range and the size of the significant coherence area between the cortex and different muscles were different. The significant coherence frequency range between the CZ channel and GM was mainly concentrated from 22 to 27 Hz, while the significant coherence frequency range with TA was mainly concentrated from 17 to 23 Hz. In addition, the coherence between the CZ channel and GM was greater than that between the CZ channel and TA.

### 3.2. Bidirectional Transfer Entropy between the Cortex and Muscles

**Figure 5:**
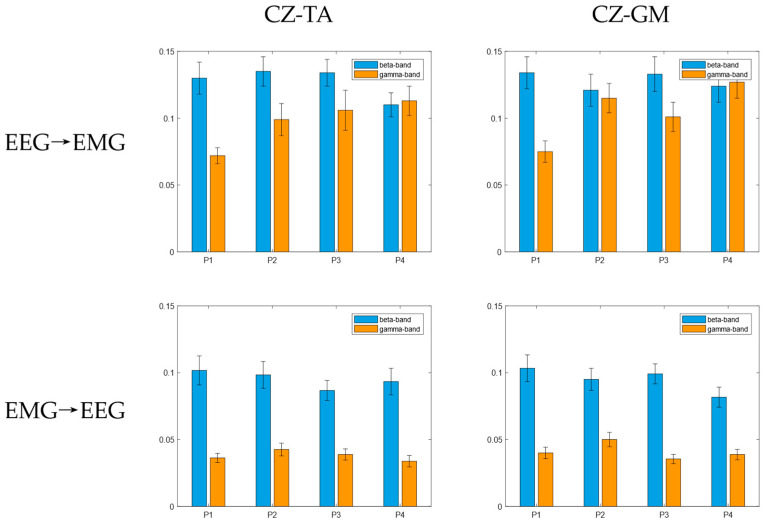
The mean and standard deviation of bidirectional TE.

On the basis of CMC analysis, the EEG and EMG data were filtered into the beta band and the gamma band. The mean TE from cortex to muscle (EEG → EMG) and from muscle to cortex (EMG → EEG) in the beta band and the gamma band under the four balance paradigms is shown in Figure 5. It can be seen that there is indeed a bidirectional coupling relationship between the cerebral cortex and the muscles of the lower limbs, forming a sensorimotor control loop. Regardless of the paradigm, the mean value of TE from EEG to EMG is greater than that from EMG to EEG in both the beta band and gamma band. In the four balance tasks, the coupling value of the cortex and muscles in the beta band was the largest in both the ascending neural pathway and the descending neural pathway. 

### 3.3. Functional Brain Network Characteristics

**Figure 6:**
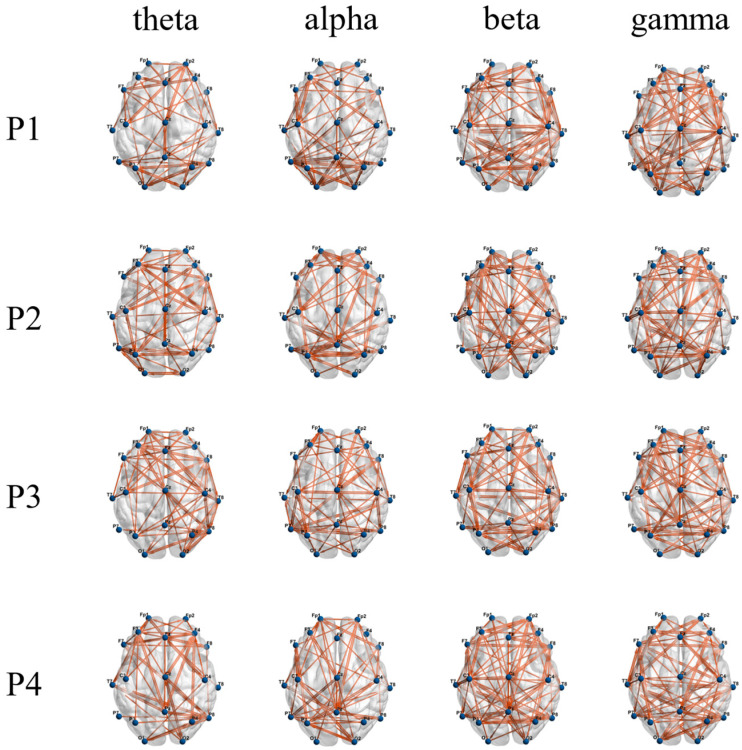
Display of functional brain networks in each frequency band under different balance paradigms. The blue node indicates the brain network channel; the orange line indicates bidirectional information flow between network nodes.

**Figure 7:**
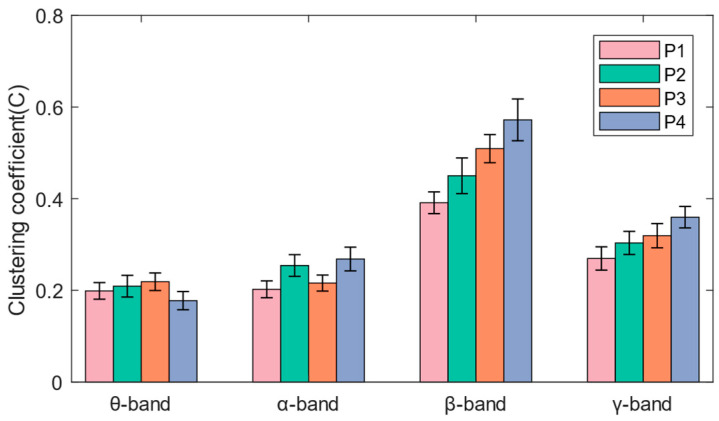
The mean and standard deviation of the average clustering coefficients for each band under the four paradigms.

As shown in Figure 6, the network connections of the theta band and alpha band are concentrated in the frontal and occipital regions, while the beta band and gamma band also have significant network connections in the central region. It can be seen in Figure 7 that when subjects performed balance paradigms of different difficulty, the mean of the *C* of the beta band was the largest, followed by that of the gamma band. As the difficulty of the balance paradigm increased, the *C* of the beta band significantly increased (* *p* < 0.05).

**Figure 8:**
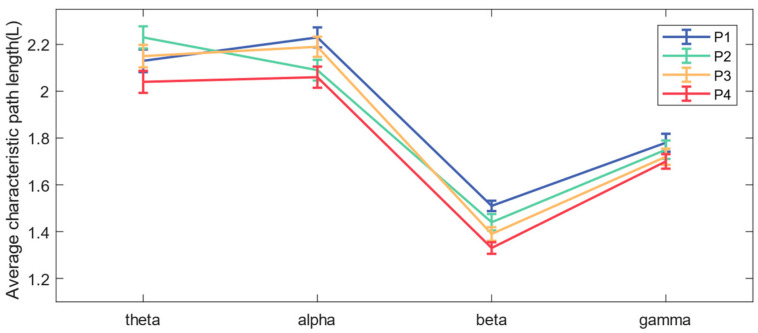
The mean and standard deviation of the average characteristic path length for each band under the four paradigms.

In addition, comparing the mean of the functional brain network *L* in each frequency band under the four balance paradigms in Figure 8, we concluded that the mean *L* in the beta band was the smallest, while the *L* of the beta band decreased significantly (* *p* < 0.05) as the difficulty of the balance paradigm increased.

## 4. Discussion

In this study, we explored the neurophysiological activities and regulation mechanism of standing balance by quantifying the changes in cortico-muscular coupling and brain network topology. Existing research has found that when subjects perform lower limb movement tasks, the central part of the primary motor cortex (corresponding to CZ) is significantly activated. Therefore, this paper focused on the cortico-muscular coupling between CZ and the lower limb muscles [28]. It was found that CMC, causality, and functional brain network characteristics were altered under the four balance paradigms.

In this paper, the mean of the significant coherence area of the 10 subjects in the beta band and the gamma band was calculated. The results showed that the subjects showed more significant CMC in the beta band when performing all four balance paradigms, which indicated that the neural oscillations between the cortex and the lower limb muscles mainly occurred in this frequency band when subjects maintained standing balance. In addition, the significant coherence areas of the beta band and gamma band showed different changes. Some scholars have found that when subjects perform a stable static force output task, CMC mainly appears in the beta band; when subjects perform a dynamic force output task, CMC shifts to the gamma band [29]. Under P1, the human body was in a state of good balance and could maintain posture stability only by relying on the static contraction of the lower limb muscles. As the difficulty of balance increased, subjects could not rely solely on the static contraction of muscles to maintain stability. They also needed to dynamically adjust their posture with the ankle joint as the pivot, which may have led to a shift in CMC towards the gamma band. Compared with single sensory input blockades, when subjects lost both vision and proprioception while maintaining standing balance, the central nervous system needed to strengthen the level of neural regulation, which caused a stronger resonance of the sensorimotor system in the gamma band. Moreover, there were differences in the significant coherence frequency range and the size of the significant coherence area between the cerebral cortex and different lower limb muscles. These phenomena are related to the different activation patterns of muscles caused by differences in people’s movements in daily life [30,31]. The above results indicate that CMC can reflect the physiological mechanism of neural control and muscle activation during standing balance.

Although CMC is of obvious value in cortico-muscular synergy analysis, it cannot be used to describe and infer the direction of information flow. The results in this paper showed that the mean TE of each frequency band in the EEG → EMG direction under the four balance paradigms was greater than that in the EMG → EEG direction. This not only shows that there is a bidirectional information flow between the cerebral cortex and lower limb muscles when the human body maintains standing balance but also shows that the cortico-muscular coupling is stronger in the descending neural pathway. During balance regulation, the cerebral cortex needs to recruit motor units in the muscles. At the same time, muscle contraction transmits feedback information to the central nervous system. Through this bidirectional mode of information interaction, the expected motor control is accomplished more precisely. The brain is the command center of the entire motor system, controlling motor behavior through complex neural pathways [32], which may result in a higher connection strength of the descending neural pathway. Furthermore, the cortex and muscles showed higher causality in the EMG → EEG direction in the beta band when the subjects performed the four balance paradigms, which is consistent with the research results of Liu et al. [33] and Cheng et al. [34], indicating that muscles may transmit information back to the nerve center through the beta band.

In this paper, functional brain networks were constructed based on TE between 19 EEG channels, and network characteristics were calculated. It was found that network connections of different frequency bands were concentrated in different brain regions, which may be the result of functional brain differentiation and complex network properties [35]. It has been confirmed that when the brain performs more difficult cognitive tasks, the brain network will show higher global efficiency [36]. The increase in the average clustering coefficient and the decrease in the average characteristic path length both indicate that the efficiency of brain network information processing is improved [37]. The experimental results of the P1–P4 paradigms in this paper showed that as balance difficulty increased, the *C* of the beta band increased significantly, and *L* decreased significantly. Due to the increased difficulty of balance, the cerebral cortex needed to mobilize more sensorimotor resources and concentration, and the information processing ability of the entire brain area in the beta band was enhanced. The brain network in the beta band exhibited the highest global efficiency, with the strongest interactions and synchronous activity between brain regions. In addition, brain network activity in the alpha band showed higher global efficiency during P2 and P4 than during P1 and P3, which may be related to the influence of visual stimulation on alpha wave oscillation [38].

This paper has achieved some meaningful results in the study of the cortico-muscular coupling under different standing balance paradigms, but there are also certain limitations. First, the number of participants was limited and their gender distribution was uneven. Second, all experimental participants were young adults. Osteoporosis and muscle atrophy due to aging may cause changes in the neural mechanisms of balance control. Finally, the subjects in this paper were all healthy; in the future, experiments need to be conducted on patients with movement disorders or cognitive impairments.

## 5. Conclusions

In short, under the four balance paradigms designed in this paper, the CMC of the beta band is the most significant. The CMC of the gamma band increases with increasing balance difficulty, which suggests a change in the sensorimotor system’s balance planning. In this study of cortico-muscular bidirectional coupling, TE in the EEG → EMG direction in each frequency band was greater than that in the EMG → EEG direction. Moreover, in both the ascending and descending neural pathways, the beta band had higher TE values, which indicates that the beta band is more crucial for cortical–muscular interaction. The functional brain network of the beta band had the highest global efficiency under all four balance paradigms; similarly, the functional brain network of the beta band showed better topological structures as balance difficulty increased. This suggests that beta waves may be related to the integration of sensorimotor resources. The above results indicate that under different balance paradigms, the cortico-muscular coupling relationship and functional brain network will exhibit distinct characteristics. Information from this study may be used for the assessment of balance rehabilitation in the future.

## Figures and Tables

**Table 1:** Experimental paradigms.

Paradigms	Details
P1	Normal perception
P2	Vision is blocked (eyes closed)
P3	Proprioception is blocked (sponge pad under feet)
P4	Both vision and proprioception are blocked (eyes closed, sponge pad under feet)

**Table 2:** Statistics of the means of the TA and GM significant coherence areas (×10^−3^).

	CZ-TA		CZ-GM	
	Beta	Gamma	Beta	Gamma
P1	30	3	37	5
P2	21	7	29	10
P3	24	9	24	12
P4	20	14	26	16

## Data Availability

The data presented in this study are available on request from the corresponding author. The data are not publicly available due to the need to protect the privacy of the subjects.
